# The Christian Advocate and the Keeley Cure

**Published:** 1895-06

**Authors:** 


### THE *CHRISTIAN ADVOCATE* AND THE KEELEY CURE.

Several months ago the New York *Christian Advocate*, edited by Rev. J. M. Buckley, D.D., undertook to investigate the claims of the much-advertised Keeley Cure for drunkenness. The object of this investigation, as stated by Dr. Buckley, was "not to prove the system worthless, or to deter drunkards hopeless of restoration by other means from trying it; but simply to ascertain whether the assertion of its inventor and his agents, that ninety-five per cent. o all cases treated are cured, is true."

The statement had been repeatedly made by Keeley and his admirers that more than two hundred thousand persons in the United States had taken this treatment for inebriety; that ninety-five per cent. of them had been cured and that five per cent. would be a large allowance to make for relapses.

The *Advocate* solicited information from the physicians and ministers among its subscribers, giving their personal observations among those who were known to have tried the Keeley method. The investigation has been conducted fairly and only from the desire to know the truth. We now have the result. There were ninety-two responses to this invitation, representing sixty-eight ministers and twenty-four physicians. Letters came from twenty-seven States and Canada, only one being allowed from the same locality. These letters report on 534 graduates of Keeley institutions; 275 are reported cured; 251 relapsed; 13 became insane, 11 died and 2 committed suicide. Percentage of relapses, 51½. This seems to dispose effectually of the claim of Keeley that not more than five per cent. relapse. Dr. Buckley states the matter mildly when he concludes that Keeley is pursuing quackish methods and that he is governed primarily by the desire to make as much money as he can, and hence the chief reason for keeping his remedies secret.

These investigations and results will not occasion any surprise among medical men, and the latter will be the first to commend and sympathize with the spirit in which they were conceived and conducted. We take pleasure in referring the reader to Dr. Buckley's excellent editorial in the *Christian Advocate*, May 2d.

---

WE call attention to our New York letter in this issue upon the diphtheria antitoxin. This was prepared at our special request by a very capable and conservative practitioner in New York, and his views are eminently entitled to weight and consideration. Dr. Ludlow will contribute other papers to THE JOURNAL.

---

CONGRESS has granted permission to the American Surgical Association and the Alumni Association of Jefferson Medical College to erect a statue in Washington in memory of the late Dr. Samuel D. Gross.

